# Bidirectional two-sample Mendelian randomization study of atrial fibrillation and breast cancer

**DOI:** 10.3389/fcvm.2024.1434963

**Published:** 2024-11-25

**Authors:** Fan Ding, Chen Chen, Yuling Wang, Tingting Zhu, Linke Jiao, Zihuan Shen, Zhiwei Zhang, Lifei Lv, Xiangning Cui, Yingdong Lu

**Affiliations:** ^1^ Department of Cardiovascular Unit, Guang’anmen Hospital, China Academy of Chinese Medical Sciences, Beijing, China; ^2^ Graduate School of Beijing University of Chinese Medicine, Beijing, China

**Keywords:** atrial fibrillation, breast cancer, Mendelian randomization, meta-analysis, causal association

## Abstract

**Background:**

Observational studies have shown an association between Breast Cancer (BC) and Atrial Fibrillation (AF). However, due to confounding factors and reverse causality, the causal role between BC and AF remains unclear. In this study, bidirectional two-sample Mendelian randomization (MR) combined with meta-analysis was used to evaluate the causal association between BC and AF.

**Methods:**

Based on the Genome-Wide Association Studies (GWAS) summary data sets, the Inverse variance weighted (IVW) method was used as the main method, the weighted median method and MR-Egger method were used for Bidirectional Two-Sample Mendelian Randomization, and the Egger intercept test was used to detect horizontal pleiotropy. Heterogeneity was tested by Cochran's Q test, and sensitivity analysis was performed by “leave-one-out”. GWAS data for AF and BC were obtained from three separate databases (FinnGen, UKBiobank, GWAScatalog) for European individuals. Finally, meta-analysis was performed on the MR Analysis results from different databases.

**Results:**

The pooled IVW results showed no evidence of an effect of BC on the risk of AF (IVW: OR = 0.9994; 95% CI = 0.9967–1.0022). There was also no evidence of an effect of AF on BC risk (IVW: OR = 0.9970; 95% CI = 0.9154–1.0859).

**Conclusion:**

The results of the Bidirectional Two-Sample Mendelian Randomization study show that there is no causal relationship between BC and AF.

## 1 Introduction

With the increase in global average life expectancy and the survival time of chronic diseases, the prevalence of Atrial Fibrillation (AF) is on the rise worldwide (1). According to the data from the FSH (Framingham Heart Study), the prevalence of AF has increased 3-fold in the last 50 years (2). AF is directly associated with increased mortality, and patients with AF do not die directly from the arrhythmia, but from the accompanying comorbidities and complications (3). In addition to cardiovascular complications such as heart failure, stroke, and systemic embolism, AF patients also have a significantly increased risk of non-cardiovascular diseases, among which cancer is the main cause of non-cardiovascular death (4). Especially in thoracic cancer, AF may be induced by cardiac infiltration, inflammation, mechanical interference, radiotherapy, cytotoxic therapy, targeted therapy, and other factors (5). Breast cancer (BC) is one of the most common malignant tumors in the chest. The incidence of BC is increasing year by year, and there is a trend of younger patients. Breast cancer has a high incidence, strong invasion, and easy recurrence and metastasis, which seriously affects the survival rate and quality of life of patients (6).

It has been clinically observed that BC and AF often occur simultaneously (7, 8). Current studies have found that there may be a correlation between them, but the results of existing studies are not very consistent. A SEER-Medicare analysis study showed that the incidence of AF was significantly higher after BC diagnosis, and the higher the cancer stage at diagnosis, the higher the risk of AF. Moreover, patients with new onset AF after the diagnosis of cancer have a higher 1-year mortality rate, which is mainly caused by cardiovascular diseases and has nothing to do with cancer (9). Some systematic reviews and meta-analysis studies have found that BC can increase the risk of AF (7), and some meta-analyses have shown that there is a bidirectional association between the two: the risk of AF in BC patients increases by 43%, and the risk of BC in AF patients increases by 18% (10). However, a cohort study involving the Danish population showed that BC was not associated with the occurrence of AF (4). Other studies have shown a reduction in 10-year cardiovascular mortality in BC patients (11). These contradictory results may be due to confounding factors and reverse causality. Therefore, the causal effect between BC and AF is not very clear at present.

Mendelian Randomization (MR) is an analysis of genetic variables that follows Mendelian laws of heredity, using single-nucleotide polymorphisms (SNP) as an instrumental variable (IVs) to assess causal associations between exposure factors and outcome variables (12). Since the genetic variation in MR follows the principle of random allocation of alleles to offspring, it is not susceptible to confounding factors and reverse causality (13, 14). Genetic methods can be an effective alternative to assess relationships when it is not feasible to test causality in randomized controlled trials or observational studies that provide biased associations due to potential confounders or reverse causality. Therefore, this study used the genome-wide association study (GWAS) data set to analyze the causal relationship between BC and AF through a Bidirectional Two -Sample Mendelian Randomization study. As the conclusions of IVs from different database sources are not consistent, the results of different databases are analyzed by using a fixed effect model or random effect model to obtain more credible conclusions (15).

## 2 Materials and methods

### 2.1 Study design

**Figure 1:**
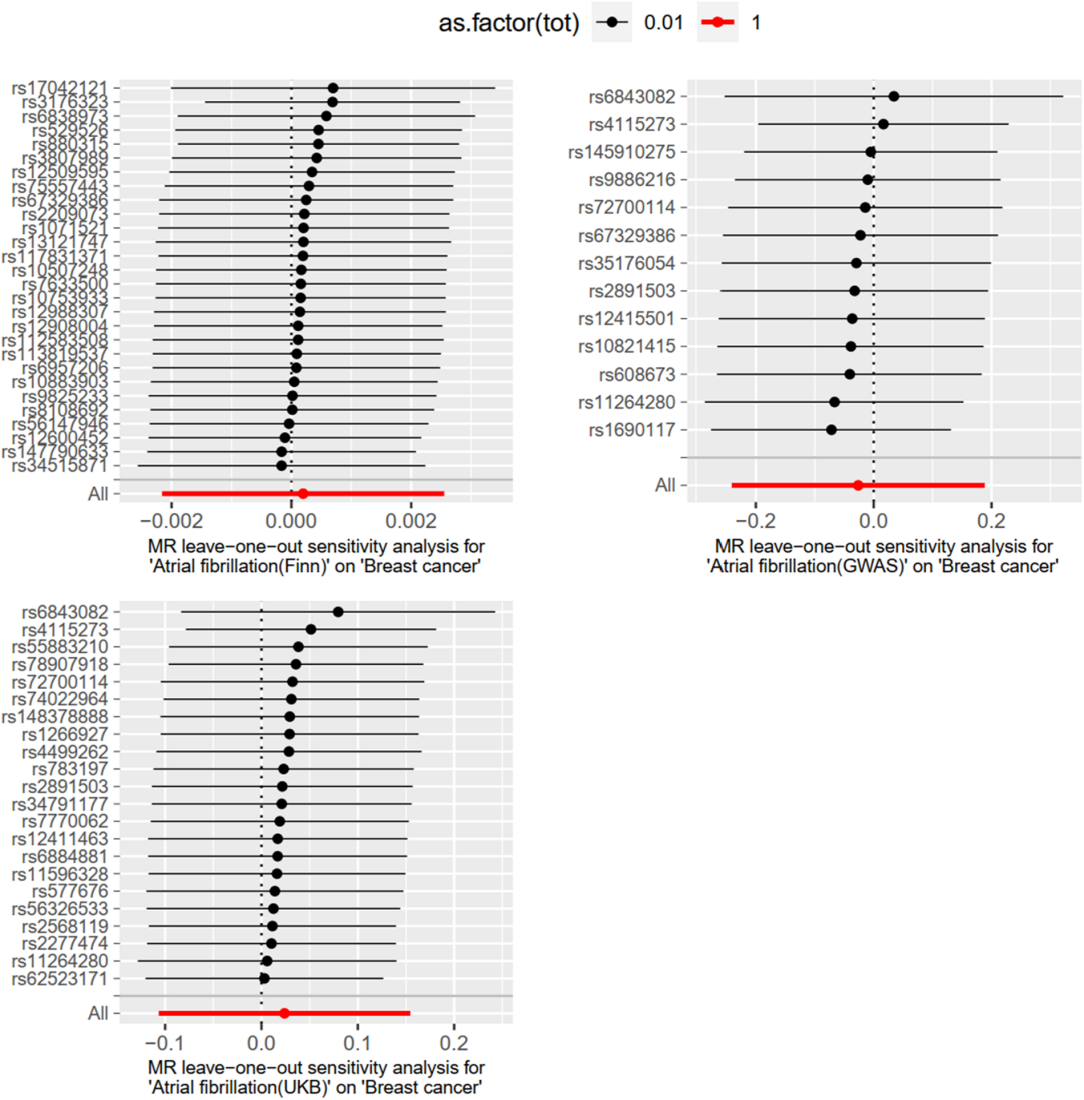
Sensitivity analysis was performed using “leave-one-out”.

We used bidirectional two-sample MR to assess the causal relationship between BC and AF, and the study design is shown in Figure 1. In this study, BC was used as the exposure factor and AF as the outcome variable, and a two-sample MR Analysis was used. SNPs significantly associated with BC were obtained as IVs from FinnGen, UKBiobank, and GWAScatalog databases, respectively. Egger intercept test was used to detect horizontal pleiotropy, and Cochran's Q method was used to test heterogeneity. Sensitivity analysis was performed by “leave-one-out” to verify its reliability, and then the MR Results obtained from the analysis of different databases were subjected to meta-analysis. Then AF was used as the exposure factor and BC as the outcome variable. SNPs significantly associated with AF were also obtained from FinnGen, UKBiobank, and GWAScatalog databases as IVs, and the above steps were repeated to finally conclude.

To better assess causal effects, SNPs screened as IVs must satisfy three assumptions (16): (1) Genetic variation as an instrumental variable must be strongly correlated with exposure; (2) Genetic variation was not associated with confounding factors of exposure and outcome; (3) Genetic variation could affect the outcome only through exposure, but not through other pathways.

### 2.2 Data sources

This study related to the GWAS data sets is from IEU OpenGWAS (17) (https://gwas.mrcieu.ac.uk/) independent European population summary data. Summary data for AF and BC were obtained from three separate databases: FinnGen database, UKBiobank, and GWAScatalog. For outcome data, GWAS data for BC were analyzed using the UK-biobank GWAS of a European sample of 35,102 cases and 423,458 controls (https://gwas.mrcieu.ac.uk/datasets/ukb-b-13584/). The GWAS data for AF were derived from the UK-biobank GWAS analysis of a European sample of 6,900 cases and 456,110 controls (https://gwas.mrcieu.ac.uk/datasets/ukb-b-6217/) (Table 1)*.* Because this study was based on published GWAS summary statistics, no additional ethical approval or informed consent was required.

**Table 1:** summary of the GWAS included in the study.

	Database	ncase	ncontrol	Sample size	Number of SNPs	Population	year
BC	FinnGen	1,131	122,448	423,458	16,379,784	European	2021
	UK Biobank	35,102	388,356	423,458	9,851,867	European	2018
	GWAScatalog	17,389	240,341	257,730	24,133,589	European	2021
AF	FinnGen	22,068	116,926	138,994	16,379,794	European	2021
	UK Biobank	6,900	456,110	463,010	9,851,867	European	2018
	GWAScatalog	3,537	481,061	484,598	9,587,836	European	2021

### 2.3 Screening of instrumental variables (IVs)

First, to satisfy the first MR Hypothesis that genetic variants such as IVs must be strongly associated with exposure, SNPS significantly associated with exposure factors were screened. The significant SNPs associated with exposure factors were extracted from the GWAS data of outcome variables, and the information on effect allele (EA), allele effect value (*β*), standard error (SE), and *P* value of the final instrumental variable was recorded. Second, to avoid linkage disequilibrium bias, SNPs that were significantly associated with exposure factors had to meet the following conditions: *r*^2^ < 0.001 and genetic distance of 10,000 kb (18). Third, effect allele frequencies were used to reconcile the respective exposure and outcome data sets while removing palindromic SNPS with intermediate allele frequencies. Fourth, according to MR article 2 and article 3 of the assumptions, using PhenoScanner (www.phenoscanner.medschl.cam.ac.uk), SNPS associated with confounding factors (19, 20) such as hypertension, diabetes, obstructive sleep apnea, myocardial infarction, heart failure, and smoking were removed. In addition, *F*-statistics were used to assess whether the selected instrumental variables had a weak instrumental variable bias to further verify the association hypothesis. When *F* > 10, it indicates that there is no weak instrumental variable bias (21). The *F*-statistic was calculated by the formula *F* = *R*^2^(*n* -* k* - 1)/[*K*(1 -* R*^2^)], *N* is the sample size of the exposure factor, *K* is the number of instrumental variables, and *R*^2^ reflects the degree to which the instrumental variables explain the exposure.

### 2.4 MR analysis

R4.2.2 and R package (Two Sample MR) were used for data statistical analysis. Summary statistics for the exposure and clinical outcome data sets were harmonized so that the SNP effect on the exposure and the SNP effect on the clinical outcome corresponded to the same alleles, while palindromic SNPS with intermediate alleles were removed.

Inverse variance weighting (IVW), weighted median method, MR-Egger regression method, simple mode method, and weighted mode method were used for Bidirectional Two-Sample MR to infer causality. IVW was used as the main MR Analysis method. IVW combined with the MR Effect estimation of each SNP to obtain the overall weighted estimate of the potential causal effect (22). IVW analysis results are most reliable when there is no horizontal pleiotropy of instrumental variables (23). The weighted median approach yields consistent estimates of causal effects even when up to 50% of the information comes from genetic variation in invalid instrumental variables. MR-Egger regression was used to confirm the existence of horizontal pleiotropy of instrumental variables, and the effect estimate of horizontal pleiotropy was expressed as an intercept (24). When there is horizontal pleiotropy of IVs, the MR-Egger regression method can still obtain unbiased estimates of causality (25). Heterogeneity was quantified by Cochran's Q test, with *P* < 0.05 indicating the presence of heterogeneity. If there is heterogeneity among instrumental variables, then multiplicative random effects IVW (IVW-MRE) is used to assess causal effects (26, 27). Sensitivity analysis was performed using “leave-one-out” to clarify the effect of a single SNP on the outcome by removing SNPS one by one and calculating the remaining combined effect, to verify the robustness of the results. The test level *α* was 0.05, and *P* < 0.05 was considered statistically significant.

After conducting MR analyses using various databases from each direction, the MR results were compiled and detailed in Supplementary Material 4, which encompasses the number of cases and the number of controls, OR values, *P*-values, and so forth. Based on these data, *β* values and se were calculated. Subsequently, according to the heterogeneity observed, a fixed-effects model or a random-effects model was selected to combine each result, and meta-analysis was performed using R version 4.2.2 and the R package (Meta).

## 3 Results

### 3.1 Effect of BC on AF

#### 3.1.1 Basic information of SNPs

In the analysis of the association between BC and AF, the significance threshold of BC should be relaxed appropriately to avoid inaccurate results due to too few SNPS. Firstly, 11 SNPs were screened from the FinnGen database using 1 × 10^−5^ (28) as the screening condition, and one SNP with an F-statistic less than 10 was removed. Then, the SNPS related to confounding factors were removed by query on the PhenoScanner website. The outcome database with the largest sample size was selected as the UKBiobank database, from which outcome data were extracted. Exposure and outcome data were reconciled using effect allele frequencies while palindromic SNPS with intermediate alleles were removed, leaving a total of five SNPS. Then 23 SNPs were selected from the UKBiobank database using 1 × 10^−6^ (29, 30) as the screening condition, and the calculated *F* statistics were all greater than 10. The outcome data were also extracted from the UKBiobank database, and a total of 21 SNPs were retained after the coordination and removal of palindrome sequences. Finally, 72 SNPs were extracted from the GWAScatalog database with 1 × 10^−6^ (29, 30) as the screening condition, and the *F* statistics were all greater than 10. The outcome data were also extracted from the UKBiobank database, and a total of 64 SNPs were preserved after data harmonization.

#### 3.1.2 MR analysis of BC and AF

**Figure 2:**
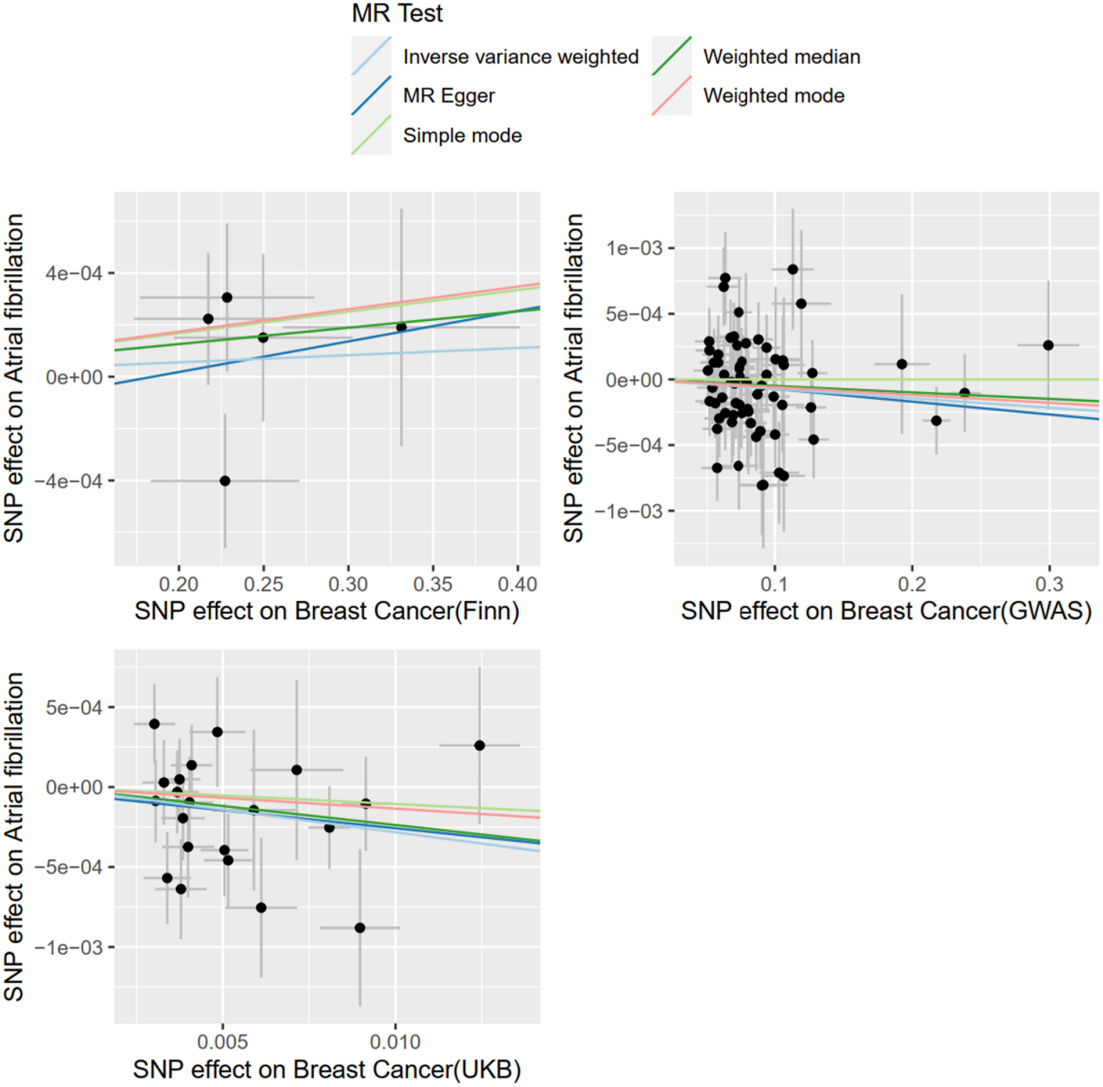
Results of MR Analysis of AF by BC.

With BC as exposure and AF as the outcome, SNP analysis extracted from the FinnGen database showed that BC was not associated with the risk of AF (OR = 1.0002; 95% CI = .9991–1.0014, *P* = 0.6357). The SNPS extracted from the UKBiobank database were analyzed by IVW method and showed a causal relationship between BC and AF risk (OR = 0.9721; 95% CI = 0.9476–0.9972) and were protective factors. The SNPS extracted from the GWAScatalog database were analyzed by IVW method and showed no causal relationship between BC and AF risk (OR = 0.9993; 95% CI = 0.9984–1.0001). The results of MR Egger and Weighted median are consistent with IVW, as shown in Figure 2, and details of the data are provided in the Supplementary Material.

#### 3.1.3 Meta-analysis results of AF on BC

**Figure 3:**
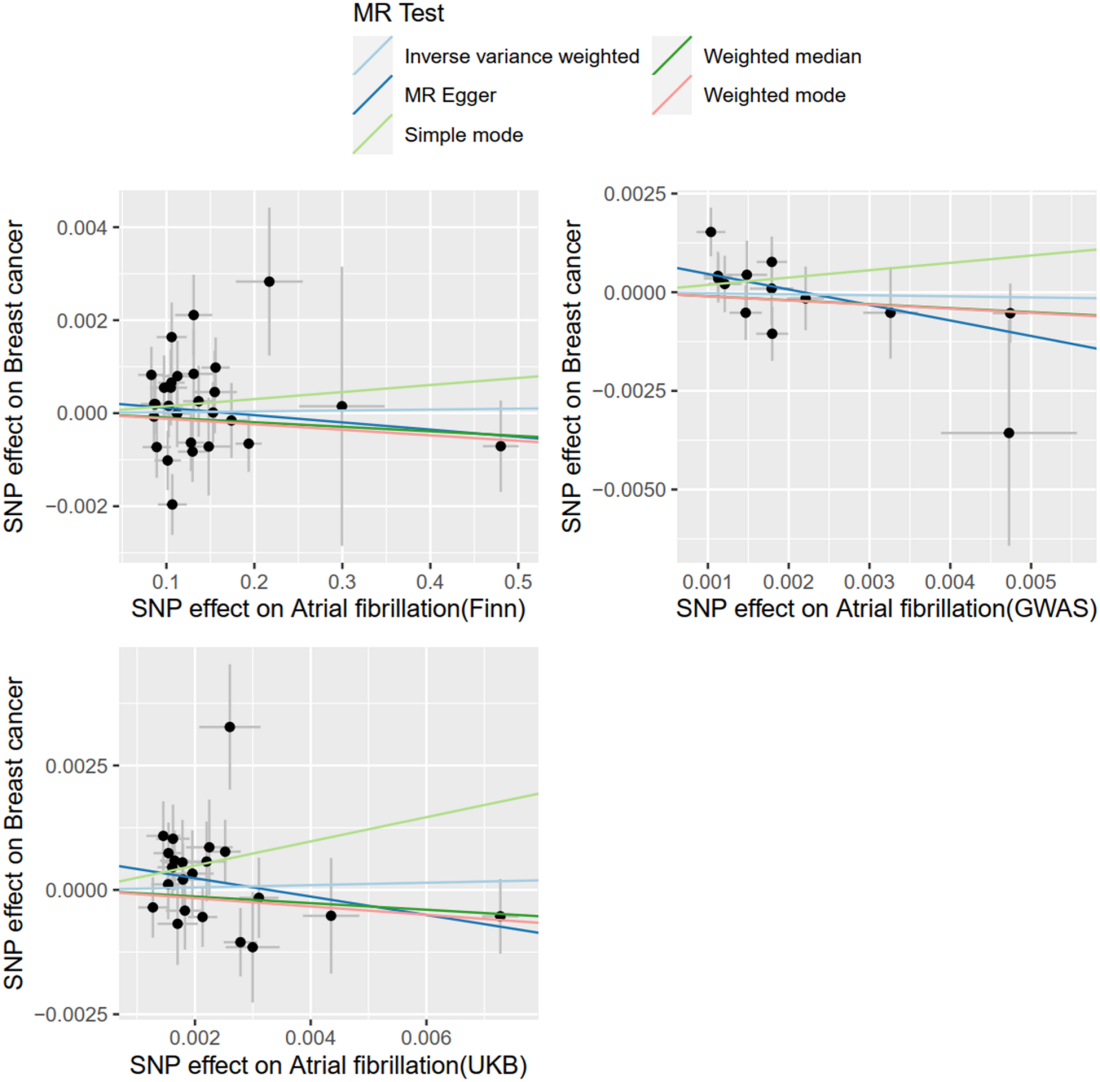
Results of MR Analysis of BC by AF.

In the MR analysis with BC as the exposure factor and AF as the outcome, given the variability in the results obtained from different databases, we conducted a meta-analysis to synthesize these research findings. Considering that *I*^2^ = 69%, suggesting substantial heterogeneity in the data, a random effects model was chosen for the analysis. The results showed an OR of 0.9994 (95% CI: 0.9967 to 1.0022), as illustrated in Figure 3. Therefore, it can be concluded that there is no causal association between AF and BC.

#### 3.1.4 Heterogeneity test and sensitivity analysis

**Figure 4:**
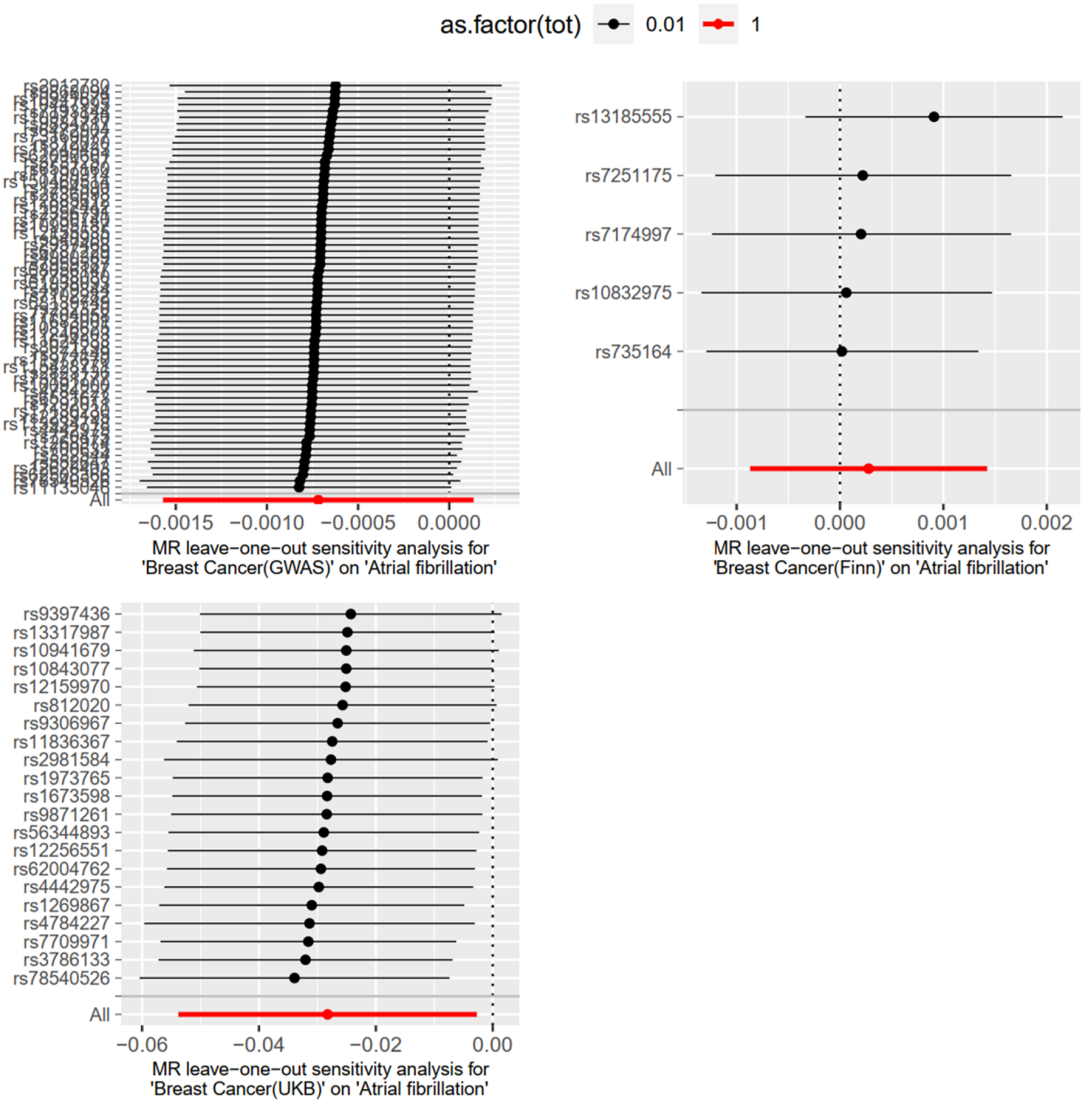
Sensitivity analysis was performed using “leave-one-out”.

Level pleiotropy was assessed using MR-Egger, while Cochran's Q was employed to test for heterogeneity. The results presented in Table 2 demonstrate that no heterogeneity or level of pleiotropy was observed among the SNPs sourced from the three database origins, thereby confirming the robustness of the MR analysis findings. The “leave-one-out” sensitivity analysis was used to test the effect of each SNP extracted from the FinnGen, UKBiobank, and GWAScatalog databases on the overall causal association. No significant differences were observed in the above causal associations when the MR Analysis was repeated after removing a single SNP. The specific results are shown in Figure 4.

**Table 2:** Heterogeneity test and sensitivity analysis of the results of MR analysis.

Exposure: BC Outcome: AF		FinnGen	UK Biobank	GWAScatalog
IVs		5	21	64
Heterogeneity test (MR-Egger)	Cochran's Q	4.4087	21.0520	74.1672
	Q_df	3	19	62
	P	0.2206	0.3339	0.1384
Heterogeneity test (IVW)	Cochran's Q	4.4520	21.0895	74.2592
	Q_df	4	20	63
	P	0.3483	0.3919	0.1569
Horizontal pleiotropy test (MR-Egger)	Intercept	−0.0002	−3.25104e-05	2.675326e-05
	P	0.875	0.856	0.7837

### 3.2 Causal effects of AF on BC

#### 3.2.1 Basic information of SNPs

With AF as the exposure factor and BC as the outcome data, and *P* < 5e^−8^ as the screening condition, 16 SNPs were extracted from the GWAScatalog, and the *F* statistics of all SNPs were calculated to be greater than 10. The UKbiobank breast cancer database with a large sample size was used as the outcome database. A total of 13 SNPs were included after the harmonization and removal of palindromic sequences. To avoid inaccurate results due to too few SNPS, 1 × 10^−6^ (29, 30) was used as the screening condition, and SNPs were extracted from the UKbiobank database and FinnGen database, respectively. UKbiobank was used as the outcome database, and the above screening steps were repeated to include 22 and 28 SNPs, respectively. Details are provided in the Supplementary Material.

#### 3.2.2 MR analysis of BC and AF

**Figure 5:**
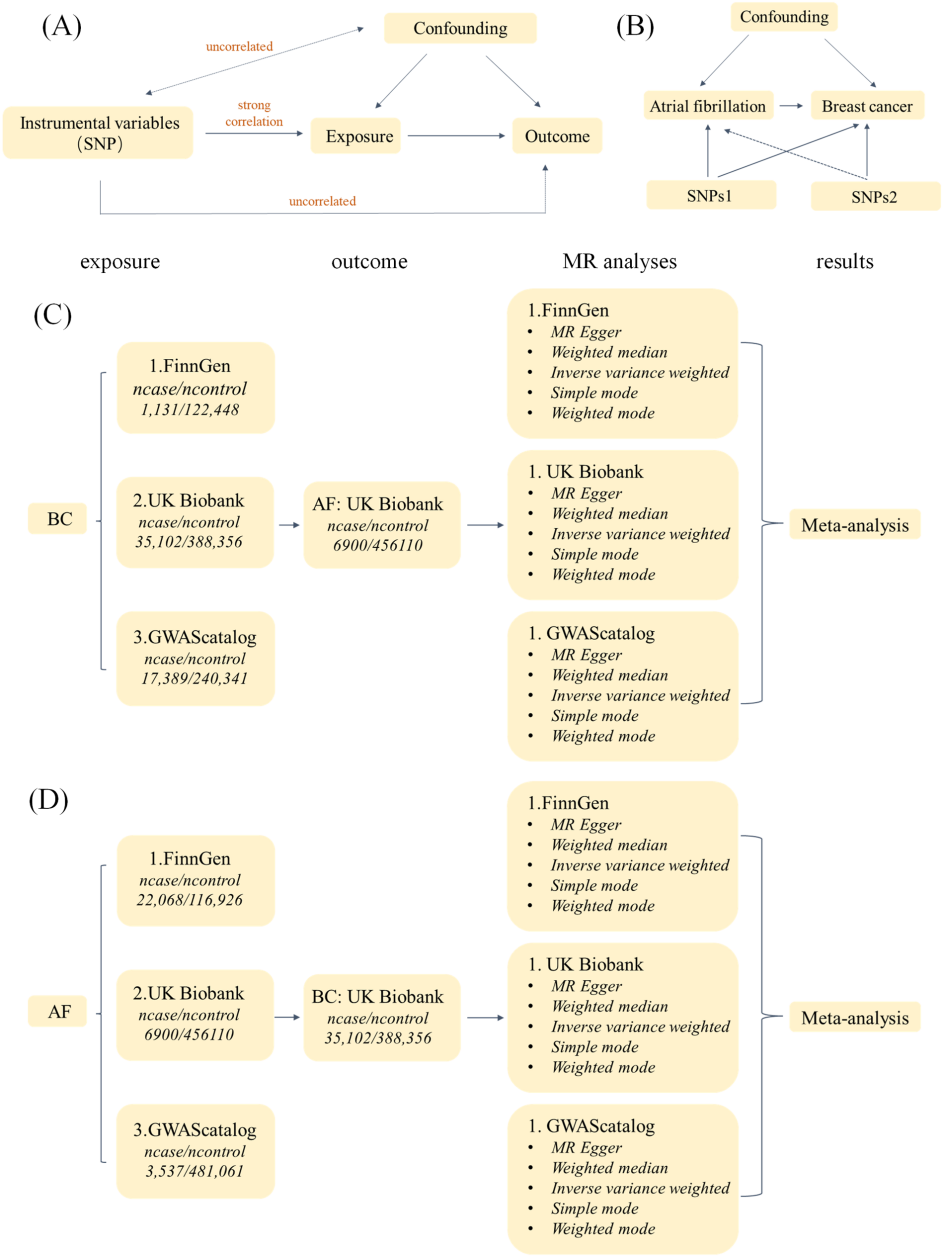
Study flow chart. **(A)** The Three Assumptions of MR Analysis. **(B)** Bidirectional two-sample MR analysis design. **(C)** Flow chart of MR Analysis with BC as exposure and AF as outcome. **(D)** Flow chart of MR Analysis with AF as exposure and BC as outcome.

With AF as the exposure and BC as the outcome, SNPS extracted from the FinnGen database were analyzed by IVW method, which showed that BC was not associated with the risk of AF (OR = 1.0001; 95% CI = .9978–1.0025, *P* = 0.8706). The SNPS extracted from the UKBiobank database were analyzed by IVW method and showed no causal relationship between BC and AF risk (OR = 1.0242; 95% CI = 0.8988–1.1671, *P* = 0.7195). The SNPS extracted from the GWAScatalog database were analyzed by IVW method and showed no causal relationship between BC and AF risk (OR = 0.9741; 95% CI = 0.7860–1.2074, *P* = 0.8109). The results of MR Egger with Weighted median were consistent with IVW, and the results are shown in Figure 5, details are provided in the Supplementary Material.

#### 3.2.3 Meta-analysis results of BC on AF

**Figure 6:**
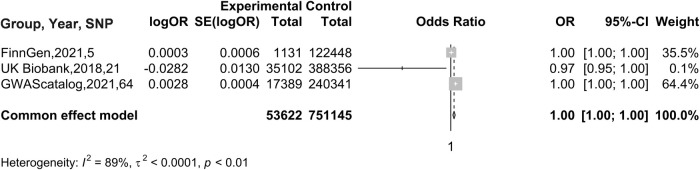
Meta-analysis results of MR Analyses from different database sources with BC as exposure and AF as outcome.

In the MR analysis with AF as the exposure and BC as the outcome, a meta-analysis was conducted to assess the MR results obtained from different databases. Since there was no heterogeneity in the data, indicated by *I*^2^ = 0%, a fixed-effects model was used for analysis. The results showed an OR of 0.9970 (95% CI: 0.9154–1.0859), as illustrated in Figure 6. Therefore, it can be concluded that there is no causal association between AF and BC.

#### 3.2.4 Heterogeneity test and sensitivity analysis

MR-Egger was used to test for level pleiotropy and Cochran's Q was used to test for heterogeneity; the results are shown in Table 3. The SNPS in FinnGen database had heterogeneity, which did not violate the core assumptions of MR, but needed to be further calculated using the IVW-MRE (multiplicative random effects) method, and the results were consistent with the IVW results. There was no heterogeneity and horizontal pleiotropy in the remaining data, and the results of the MR Analysis were robust.

**Table 3:** Heterogeneity test and sensitivity analysis of the results of MR analysis.

Exposure: AF Outcome: BC		FinnGen	UK Biobank	GWAScatalog
IVs		28	22	13
Heterogeneity test (MR-Egger)	Cochran's Q	39.8291	20.4461	9.7474
	Q_df	26	20	11
	P	0.0405	0.4303	0.5532
Heterogeneity test (IVW)	Cochran's Q	40.5301	23.9385	13.8642
	Q_df	27	21	12
	P	0.0457	0.2961	0.3095
Horizontal pleiotropy test (MR-Egger)	Intercept	0.0003	0.0006	0.0009
	P	0.5047	0.0794	0.0673

#### 3.2.3 Heterogeneity test and sensitivity analysis

**Figure 7:**
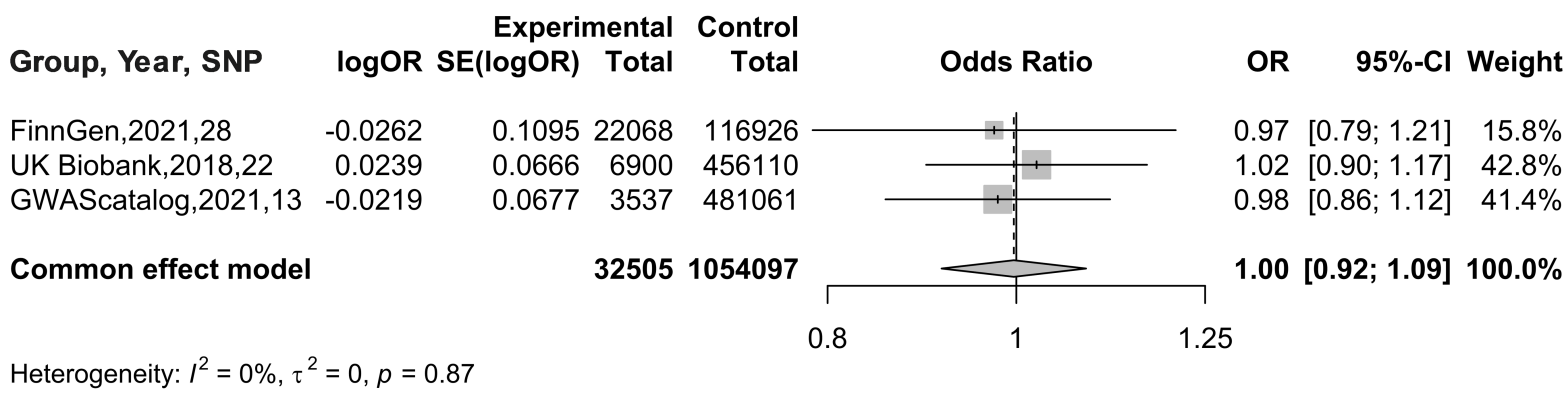
Meta-analysis results of MR Analysis from different database sources with AF as exposure and BC as outcome.

The “leave-one-out” method was used to test the influence of each SNP extracted from the FinnGen, UKBiobank, and GWAScatalog databases on the overall causal association. When a single SNP was removed and MR Analysis was repeated, no significant difference was observed in the above causal association. The specific results are shown in Figure 7.

## 4 Discussion

This study, utilizing publicly available GWAS summary data, evaluated the causal relationship between BC and AF risk through bidirectional two-sample Mendelian randomization and meta-analysis. The results showed no clear evidence of an association between BC and AF incidence, nor in reverse analyses. Sensitivity analyses confirmed the robustness of the findings.

To date, the link between BC and AF, as well as the underlying causes, has been controversial. On one hand, some studies suggest that the clinical observation of BC and AF often occurring simultaneously is indirectly due to confounding factors such as cancer and AF treatments, age, diagnostic timing, and emotional factors (31), rather than a causal relationship inherent to the diseases themselves. A cohort study in a Danish population found an increased incidence of cancer within 90 days after an AF diagnosis (4). This temporal correlation between AF diagnosis and subsequent cancer diagnosis may not be due to a causal relationship between AF itself and cancer, but rather indicative of cancer already being present at the time of AF diagnosis. Most AF cases are paroxysmal, and the probability of detecting paroxysmal AF increases with the number of patient visits. This is because during multiple medical consultations, patients may undergo multiple ECGs or other related tests, thereby increasing the opportunities for AF detection. The treatment following an AF diagnosis can also impact cancer diagnosis. For instance, anticoagulant therapy for AF may increase the risk of bleeding, leading to the discovery of cancer. Other AF-related treatments, such as amiodarone and cardiac glycosides, may also be associated with an increased risk of cancer (32, 33). Additionally, studies have found that the risk of AF significantly increases within 90 days before a cancer diagnosis, which may be related to invasive diagnostic procedures, autonomic nervous system dysfunction caused by physical pain and mental distress due to newly diagnosed cancer, and acute complications of cancer. However, the risk of AF decreases after 90 days of cancer diagnosis, possibly due to long-term anticoagulant therapy (34) and the inhibitory effect of atrial natriuretic peptide (ANP) related to AF on tumor growth (35). Furthermore, current research indicates that the severity of breast cancer, specifically its stage and grade, is associated with the development of AF. Patients with advanced (stage IV) breast cancer have more than 300% higher likelihood of developing AF compared to those with early-stage (stage I) cancer (9), which may be related to the generally poorer overall condition of patients with advanced cancer and the systemic use of chemotherapy drugs (36).

It is noteworthy that many researchers believe that cancer-related treatments play a significant role in the development of AF (37). A meta-analysis indicates that breast cancer increases the risk of AF, potentially due to the cardiotoxicity of breast cancer treatment drugs (38). Another study also found a higher relative incidence of AF in patients with stage III breast cancer undergoing chemotherapy (39)This conclusion is corroborated by a Swedish cohort study, which found that women under 60 diagnosed with invasive breast cancer (BC) in the southeast healthcare region of Sweden between 1998 and 2002 had a significantly higher risk of cardiovascular toxicity and all-cause mortality related to cancer treatment, particularly in elderly patients and those receiving anthracycline-based therapy (40). Studies suggest that anthracycline chemotherapy drugs can lead to instability and dysfunction in cardiac conduction (41). Trastuzumab can also cause reversible, non-dose-dependent cardiac dysfunction during treatment (42, 43). A study suggests that chemotherapy drugs may activate AF-related genes (44), such as docetaxel activating ANXA5 (one of the most abundant membrane-associated proteins in cardiomyocytes), thereby increasing the risk of AF. However, there is still limited understanding of the molecular mechanisms underlying chemotherapy-induced cardiotoxicity, the genetic basis of individual sensitivity to chemotherapy drugs, the interactions and mechanisms among chemotherapy drugs, and their impact on other comorbidities in cancer patients. These aspects require further attention in subsequent research. Additionally, the incidence of atrial fibrillation (AF) increases within 10 years among breast cancer patients who have undergone radiotherapy (45). A retrospective cohort study revealed a significantly elevated risk of cardiac disease related to breast cancer treatment among Asian female breast cancer patients who received adjuvant radiotherapy, even in the absence of any cardiac risk factors. This effect was found to be associated with radiation dose (46).

On the other hand, some researchers believe that there is an inherent causal relationship between BC and AF. A meta-analysis, which included 23 studies involving 8,537,551 subjects, found that the prevalence of AF among breast cancer patients was 3%, with an incidence rate of 2.7%. In the combined cohort, breast cancer patients had a 43% increased risk of developing AF, and AF patients had an 18% increased risk of developing breast cancer. Notably, this bidirectional association was not related to age, cancer treatment, or the estrogenic effects of digoxin (10).

Although the causal link between the two is controversial, there is a consensus among researchers that patients with AF and BC have increased mortality (47). A meta-analysis of 15 studies involving 2,868,010 patients with AF showed that cancer increased the incidence of bleeding events and mortality in patients with AF. Its mechanism may be related to the use of anticoagulant drugs, cancer-related bone marrow suppression, anemia, and thrombocytopenia caused by inflammation, radiotherapy, and chemotherapy (48). Among patients with AF, there is also a higher risk of stroke in patients with BC compared to those without cancer (49). Therefore, it is of great significance to clarify the etiology of AF and BC and to carry out individualized prevention and treatment as soon as possible.

The MR analysis method mimics randomized controlled trials in experimental design, yielding high-level evidence. Compared to randomized controlled trials, it offers advantages such as lower costs and larger sample sizes. Furthermore, it can effectively avoid confounding factors and reverse causal associations. Previous scholars have also used MR analysis to show no causal relationship between BC and AF (50, 51). However, we found inconsistent conclusions when conducting MR analysis using different databases. SNPs extracted from the UK Biobank and analyzed using the inverse variance weighted (IVW) method indicated a causal relationship between BC and AF risk (OR = 0.9721; 95% CI = 0.9476–0.9972). However, other databases showed no such association. Therefore, to further investigate this, we introduced a meta-analysis, integrating SNPs from three databases. Our conclusions further confirm the reliability of previous studies, and our study uses a larger sample size, effectively avoiding errors caused by individual heterogeneity. The methods used in our study also demonstrate a certain degree of innovation. The data used in our study were all derived from European population samples in GWAS databases, effectively reducing population heterogeneity. This study employed a meta-analysis to aggregate MR analysis results from different databases, making the conclusions more reliable. Additionally, multiple sensitivity analyses were conducted to verify the robustness and consistency of the results. However, our study also has certain limitations. Although no causal relationship between BC (breast cancer) and AF (atrial fibrillation) was found, a correlation between them has been observed clinically, and the underlying mechanisms require further investigation. Furthermore, since our data were all sourced from public databases, subgroup analyses based on specific factors such as age and gender were not feasible. The incidence of breast cancer is much higher in women than in men, so the bias caused by gender factors on the results may be minimal (52). However, the staging and treatment protocols of breast cancer can affect the occurrence of AF, and we were unable to perform subgroup analyses on these factors. This should be noted in subsequent research. All samples used in this study were from European populations, and the generalizability of the results to other populations remains to be validated.

## 5 Conclusion

In conclusion, this study indicates that there is no causal relationship between BC and AF, but the increased mortality rate among patients with BC and AF warrants greater attention in clinical prevention and treatment.

## Data Availability

The original contributions presented in the study are included in the article/Supplementary Material, further inquiries can be directed to the corresponding author.
